# Bacteremia by *Chryseobacterium indologenes* in a Patient with Lung Cancer: A Clinical and Microbiological Investigation

**DOI:** 10.5005/jp-journals-10071-23142

**Published:** 2019-03

**Authors:** Gitali Bhagawati, Ashutosh Bhardwaj, Rekha Sajikumar, Sukhwinder Pal Singh, Sanjeev Prajapati

**Affiliations:** 1-5 Department of Microbiology, Dharmshila Narayana Superspeciality Hospital, Delhi, India

**Keywords:** *Chryseobacterium indologenes*, Multidrug resistant (MDR), Lung cancer

## Abstract

We present a case of bacteremia by an unsual, instrinsically multidrug resistant organism, *Chryseobacterium indologenes* in a 59 year old gentleman with squamous cell carcinoma of lung with multiple metastasis. Despite of treating as per sensitivity report after isolating*Chryseobacterium indologenes*, patient could not be survived. The pathogenicity and predictability of the organism towards antibiotics, both*in vivo* and *in vitro* needs further research.

**How to cite this article:**

Bhagawati G, Bhardwaj A *et al*. Bacteremia by *Chryseobacterium Indologenes* in a Patient with Lung Cancer: A Clinical and Microbiological Investigation. Indian J Crit Care Med 2019;23(3):157-159.

## INTRODUCTION

*Chryseobacterium* *indologenes* (previously classified as *Flavobacterium indologenes*), a Gram-negative rod is an environmental organism. Infection by this organism is usually rare; however, reported cases are there causing serious infections in immunosuppressed patients from various parts of the globe^1,2^. The troublesome fact with this organism is that it presents a high rate of natural resistance against broad-spectrum cefem compounds including carbapenems^2^.

### Case Report

This document reports a 59-year-old gentleman diagnosed with moderately differentiated squamous cell carcinoma (SCC) of left lung with multiple metastasis in liver, brain, bone, subcutaneous tissue in chest and back, left adrenal gland, lymph nodes including right pulmonary hilar, mediastinal, bilateral axillary and right cardiophrenic angle. Tumour marker cytokeratin (CK)7 was found to be positive. He was admitted in ward with complains of loss of appetite and generalized weakness for 3-4 days. Patient was planned for palliative external beam radiotherapy (EBRT) to address painful bony metastasis followed by systemic chemotherapy. Blood was transfused (1 unit packed red blood cells) on the day of admission in view of low hemoglobin (7 gm/dL). Patient also had complaints of urinary retention, but due to resistance during Foley's catheterisation attempts failed and therefore cystostomy had to be done. On 3rd day of admission, patient was shifted to medical intensive care unit (ICU) due to low Glasgow Coma Scale (GCS). Central venous line (CVP) insertion was done on the same day. Investigations revealed high TLC, thrombocytopenia, dyselectrolytemia including hypernatremia, hypokalemia, deranged Kidney function test (KFT). After admission to ICU, blood and urine samples were sent for culture. Both blood and urine cultures showed growth of multidrug resistant (MDR)*E. coli*. Patient was receiving injection cefepime-tazobactum for 10 days; injection polymyxin B for 6 days.

**Fig. 1:**
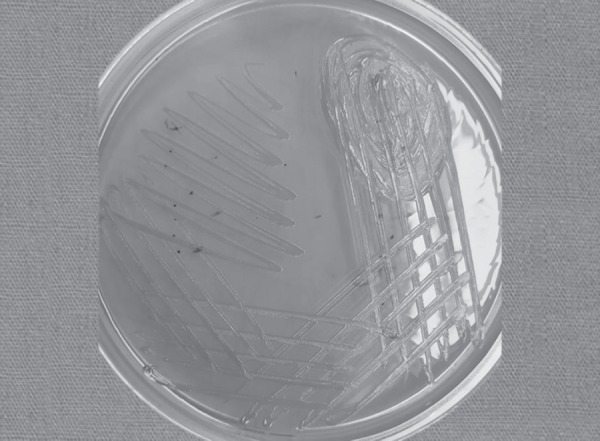
Yellowish pigmented colony of *Chryseobacterium indologenes* in MacConkey Agar media after 48 hours of incubation

After one week of stay in the ICU, repeat paired aerobic blood (right femoral line and central venous line) samples were taken in Becton Dickinson (BD) blood culture bottles and sent for culture. Repeat urine sample showed no growth. Paired set of aerobic blood culture samples were processed with the Bactec 1090 (Becton Dickinson, USA). Bacterial growth was detected within 48 hr in both bottles of the samples. Gram stain of positive blood culture bottle showed Gram-negative bacilli. Sub-cultures were done on routine Sheep Blood agar and MacConkey agar. After 24 hr of incubation, smooth, circular, yellow-pigmented colonies were grown on sheep blood agar^3^. On addition of 1 drop of 10% KOH solution, the color of the colonies was changed from yellow to red which indicates presence of flexirubin pigment. The isolate was catalase and oxidase positive, indole weakly positive and urease negative. Oxidation fermentation test results revealed oxidation positive/fermentation negative, mannitol positive non- motile organism. Final identification and sensitivity of the organism was done by Vitek 2 Compact system (BioMerieux). *Chryseobacterium indolegenes* was isolated from both the blood culture bottles. Antimicrobial susceptibility pattern of both the isolates from blood culture showed same sensitivity pattern with minimum inhibitory concentration (MIC) levels (Table 1 and Figure 1).

Patient's antibiotics were modified as per culture report and sensitivity patterns. Levofloxacin and minocycline were added and Polymyxin B discontinued. Levofloxacin was continued for 11 days while minocycline for 9 days.

**Table 1:** Antimicrobial susceptibility pattern of*Chryseobacterium indolegenes* isolated from blood culture

*Antibiotic*	*MIC*	*Interpretation*
CEFTAZIDIME	>=64	R
AMIKACIN	>=64	R
MINOCYCLINE	<=1	S
CIPROFLOXACIN	>=16	R
GENTAMYCIN	>=16	R
COLISTIN	>=16	R
CEFEPIME	>=64	R
LEVOFLOXACIN	4	I
MEROPENEM	>=16	R
AZTREONAM	>=64	R
TICARCILLIN CLAVULANIC ACID	>=128	R
IMIPENEM	>=16	R
CEFOPERAZONE-SALBACTUM	>=64	R
TRIMETHOPRIM-SULFOMETHOXAZOLE	160	R
PIPERACILLIN-TAZOBACTUM	>=128	R
TIGECYCLINE	>=8	R

On 20th day of admission in ICU, there was further deterioration of patients' general condition including sensorium and Glassgow coma scale (GCS). In view of advance nature of the disease, sepsis refractory to antibiotics, dyselectrolytemia and aspiration, the patient was put on non-invasive ventilation with informed consent from the primary responsible attendant of the patient. However, the patient had an episode of bradycardia which was followed by cardiopulmonary arrest.

## DISCUSSION

*Chryseobacterium* spp. is not usually found in human flora but widely distributed in soil, plants, food-staffs and water^4,5^. In health care institutes, water systems can act as a potential reservoir for the bacteria; thus, patients' may get colonized by this bug via various contaminated medical devices like endotracheal tube, tracheostomy tube etc.^1,4,5^ Although the pathogenicity of *C. indologenes* has not been clearly defined, biofilm production and their highly active protease have been found to be responsible for its virulence^4,6^.

In 1993, the first case of infection by *C. indologenes* was reported from a patient with ventilator associated pneumonia (VAP)^7^. During the period 1997 to 2001, SENTRY Antimicrobial Surveillance Program represented 0.03% of *Chryseobacteria* among all isolates which were responsible for bloodstream infections^2,8,9^. *Chryseobacterium spp.* usually cause infections in patients' with underlying medical illness, newborn or elderly with immunocompromising status, indwelling intravascular catheter, renal calculi long-term broad-spectrum antibiotics etc.^1,2,4,5,8,10–12^Very few case reports of infection by this organism in immunocompetent patients are available^13,14^. It can cause various infections like bacteremia, ventilator associated pneumonia (VAP), pyonephritis, biliary tract infection, lumboperitoneal shunt infection, ocular infections, burn wound infections etc.^1,2,15–18^

Some authors believe that after introduction of colistin and tigecycline, prevalence of *C.* *indologenes* infections have been increased^2,5^. The organism is intrinsically resistant to carbapenems and cephalosporins due to production of molecular Class A Beta lactamases and Class B carbapenem hydrolyzing B-lactamase. However, most active agents against this bug were found to be trimethoprim-sulfamethoxazole (TMP-SMZ) and cefoperazone- sulbactam^2^. According to the results of the SENTRY Program, ≥95% susceptibility was found with newer quinolones (garenoxacin, gatifloxacin, and levofloxacin) and TMP-SMX followed by piperacillin-tazobactam (90% susceptibility)^8,9^. Worldwide newer quinolones may represent the most appropriate antimicrobial agents against this pathogen^1^.

The present case report suggests that *C. indologenes* can cause nosocomial bacteremia in terminally ill cancer patients with other risk factors like previous MDR infections and use of broad spectrum antibiotics against them. Appropriate selection of antimicrobials is difficult despite the proper culture and sensitivity report due to unpredictable nature of the organism against the antibiotics. Further study on pathogenesis of this organism may help in future to get the proper empiric antibiotic.

## References

[1] Calderón G,, García E, (2011;). *Chryseobacterium indologenes* infection in a newborn: a case report.. Journal of Medical Case Reports..

[2] Teke T A,, Oz F N, (2014;). *Chryseobacterium indologenes* Septicemia in an Infant.. Case Reports in Infectious Diseases..

[3] Collee J.G.,, Miles R. S., (2008.). Tests for identification of bacteria. Mackie & McCartney Practical Medical Microbiology..

[4] G Bhuyar,, S Jain, (2012;). Urinary tract infection by *Chryseobacterium indologenes*.. Indian Journal of Medical Microbiology..

[5] Chen F.,, Wang G., (2013;). Clinical and epidemiological features of *Chryseobacterium indologenes* infections: analysis of 215 cases.. Journal of Microbiology, Immunology and Infection..

[6] Hsueh PR,, Teng LJ, (1996;). Clinical and microbiological characteristics of *Flavobacteriumindologenes* infections associated with indwelling devices.. Journal of Clinical Microbiology..

[7] Srinivasan G.,, Muthusamy S., (2016;). Unforeseeable presentation of *Chryseobacterium indologenes* infection in a paediatric patient.. Biomedical Central Research Notes..

[8] Christakis G.B.,, Perlorentzou S. P., (2005;). *Chryseobacteriumindologenes* non-catheter-related bacteremia in a patient with a solid tumor.. Journal of Clinical Microbiology..

[9] Kirby JT,, Sader HS, (2004;). Antimicrobial susceptibility and epidemiology of a worldwide collection of *Chryseobacterium* spp.: report from the SENTRY Antimicrobial Surveillance Program (1997-2001).. Journal of Clinical Microbiology..

[10] Hsueh P.,, Hsiue T., (1996;). *Flavobacterium indologenes* bacteremia: clinical and microbiological characteristics.. Clinical Infectious Diseases..

[11] Cascio A.,, Stassi G., (2005;). *Chryseobacterium indologenes* bacteraemia in a diabetic child.. Journal of Medical Microbiology..

[12] Alfouzan W.,, Dhar R., (2014.). Clinical and microbiological characteristics of *Chryseobacterium* spp. isolated from neonates in Kuwait.. JMM Case Reports..

[13] Cunha V.,, Ferreira M., (2014.). Community-acquired *Chryseobacterium indologenes* in an immunocompetent patient.. JMM Case Reports.

[14] McKew G.,, Bourbeau. P. (2014;). Severe Sepsis Due to *Chryseobacterium indologenes* in an Immunocompetent adventure traveler.. Journal of Clinical Microbiology..

[15] Green B. T.,, Nolan. P. E. (2001;). “Cellulitis and bacteraemia due to *Chryseobacterium indologenes*,”. Journal of Infection..

[16] Tatari H. A.,, Asmar B. I., (2007;). Lumboperitonial shunt infection due to *Chryseobacterium indologenes*.. Pediatric Infectious Disease Journal..

[17] Bayraktar M. R.,, Aktas E., (2007;). Postoperative *Chryseobacterium indologenes* bloodstream infection caused by contamination of distillate water.. Infection Control and Hospital Epidemiology..

[18] Lin YT,, Jeng YY, (2010;). Clinical and microbiological characteristics of *Chryseobacterium indologenes* bacteremia.. J Microbiol Immunol Infect..

